# Changes in remimazolam plasma concentration associated with the Pringle maneuver during hepatectomy: a pilot study

**DOI:** 10.1186/s40780-026-00597-9

**Published:** 2026-06-16

**Authors:** Shunya Ogawa, Hirotsugu Kanda, Takahiro Ito, Manabu Suno, Kazuo Matsubara, Sarah Kyuragi Luthe, Tomoyuki Kawamata

**Affiliations:** 1 Department of Anesthesiology, Wakayama Medical University School of Medicine, 811-1 Kimiidera, Wakayama, 640-8509 Japan; 2 School of Pharmaceutical Sciences, Wakayama Medical University, Wakayama, Japan

**Keywords:** Remimazolam, Pringle maneuver, Hepatectomy, Plasma concentration

## Abstract

**Background:**

Remimazolam (RMZ) is an ultrashort-acting benzodiazepine used in general anesthesia, metabolized rapidly by carboxylesterase 1, an enzyme primarily located in the liver. The Pringle maneuver (PM), an established technique commonly employed during hepatectomies to reduce bleeding, involves clamping major vessels, potentially affecting drug metabolism and clearance. Therefore, we conducted a study to investigate the changes in plasma concentration (Cp) of RMZ associated with the PM during hepatectomy and the impact on bispectral index (BIS) values.

**Methods:**

This single-center prospective observational pilot study included ten patients undergoing hepatectomy using the PM.

**Results:**

Our findings showed that the changes in RMZ Cp immediately following each PM tended to be higher than those before PM in six cases, though this was not observed in others. However, there was no statistically significant difference between the median RMZ Cp after the final PM (1099 [723–1386] ng/mL) and before the initial PM (707 [641–856] ng/mL). Similarly, the median BIS value after the final PM (45 [40–46]) was comparable to the BIS prior to initiation of PM (46 [44–50]).

**Conclusions:**

This study suggested that RMZ may be safely administered during hepatectomies with PM in patients with Child-Pugh classification A.

**Trial registration:**

Clinical trial number: not applicable.

## Background

Remimazolam (RMZ) is an ultrashort-acting benzodiazepine used in general anesthesia, producing a sedative effect by acting on the benzodiazepine-binding site of the γ-aminobutyric acid receptor [1]. RMZ is rapidly metabolized by carboxylesterase 1 (CES1), an enzyme primarily located in the liver [1, 2]. Hepatic drug clearance is determined by hepatic blood flow and intrinsic metabolic capacity, and drugs can be broadly classified as flow-limited or capacity-limited based on their hepatic extraction ratio. Drugs with high hepatic extraction ratios are primarily flow-limited and are therefore highly sensitive to changes in hepatic blood flow, whereas drugs with low extraction ratios are more dependent on enzymatic activity and are less affected by blood flow.

The Pringle maneuver (PM), a technique commonly employed during hepatectomy to reduce bleeding, involves temporary occlusion of hepatic inflow, resulting in reduced hepatic blood flow and ischemia-reperfusion injury. This intervention may affect the pharmacokinetics of various drugs, including RMZ, potentially resulting in increased plasma concentrations (Cp). Patients with severe hepatic impairment (Child-Pugh classification C) have been reported to exhibit reduced clearance of RMZ [3], likely reflecting decreased intrinsic metabolic capacity. Since RMZ is primarily metabolized by CES1, it is generally considered to be capacity-limited, although contributions from altered hepatic blood flow cannot be excluded. Furthermore, experimental studies have demonstrated that inflammatory liver injury can suppress CES1 activity [4], indicating that ischemia-reperfusion associated with the PM may reduce metabolic capacity. Transient reductions in hepatic blood flow during the PM may also contribute to altered RMZ pharmacokinetics, although the extent of this contribution remains unclear. Thus, even in patients with normal preoperative liver function, the effect of the PM on RMZ pharmacokinetics may not be negligible. Nevertheless, the extent to which these mechanisms influence RMZ pharmacokinetics remains unclear.

We conducted a pilot study to investigate changes in RMZ Cp associated with the PM during hepatectomy, as well as corresponding changes in bispectral index (BIS) values. If this pilot study demonstrates a tendency for RMZ Cp to increase during the PM, we aim to conduct a larger-scale study to elucidate the impact of this increase on BIS values and to determine the appropriate dosing of RMZ during the PM.

## Methods

The aim of this single-center prospective observational study was to investigate the changes in Cp of RMZ associated with the PM during hepatectomy and the impact on bispectral index (BIS) values. No interventions were assigned by the investigators; all aspects of clinical management, including RMZ administration and use of the PM, were determined solely by the attending physicians as part of routine clinical care. Therefore, this study does not meet the World Health Organization/International Committee of Medical Journal Editors definition of a clinical trial. This study was approved by the Wakayama Medical University Research Ethics Committee (Institutional Review Board No.3677). We recruited ten patients presenting at the Wakayama Medical University Hospital from November 2022 to March 2023 for an elective open or laparoscopic/robot-assisted hepatectomy using the PM under general anesthesia. Written informed consent was obtained from all patients. We included patients aged > 20 years with an American Society of Anesthesiologists Physical Status (ASA-PS) 1 or 2. The following patients were excluded: Child-Pugh classification B or C, obesity (body mass index > 35 kg/m2), or patients receiving benzodiazepines at the time of enrollment.

### Anesthesia

No patient was premedicated. All patients were monitored by Electrocardiography, noninvasive blood pressure, invasive arterial pressure, pulse oximetry, BIS, temperature, and a train-of-four count. General anesthesia was induced with intravenous RMZ (12 mg/kg/h). After confirming loss of consciousness, RMZ was reduced to 1 mg/kg/h, and remifentanil was started at 0.1–0.3 µg/kg/min. Endotracheal intubation was performed following muscle relaxation with rocuronium 0.6 mg/kg. An arterial catheter was inserted for invasive blood pressure monitoring and blood sampling. During surgery, RMZ was continuously infused at 1 mg/kg/h, with a target BIS range of 40–60; if signs of arousal were observed, defined as BIS values exceeding 70 for a prolonged period, a bolus dose of 0.2 mg/kg was administered at the discretion of the attending anesthesiologist. For postoperative pain management, all patients received continuous epidural analgesia with patient-controlled epidural analgesia except for one patient who received a peripheral nerve block followed by intravenous patients-controlled analgesia after completion of surgery. Following the end of surgery, RMZ was discontinued, and all patients were extubated.

### Pringle maneuver and blood sampling

**Fig. 1:**
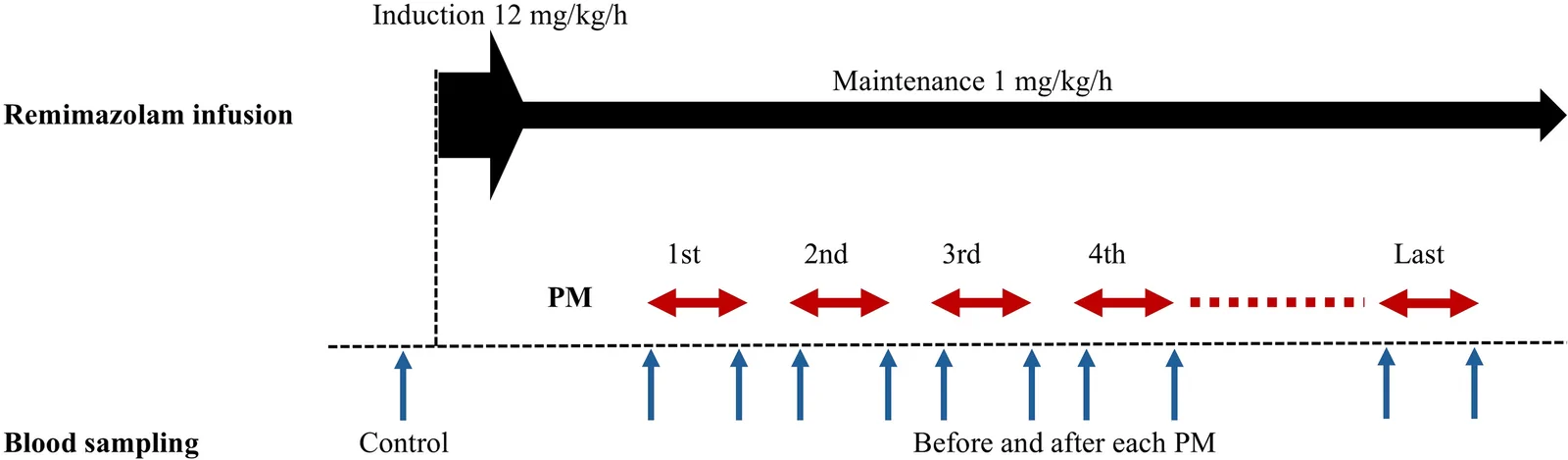
Study protocol. Remimazolam (RMZ) was initially infused at 12 mg/kg/h until loss of consciousness and subsequently maintained at 1 mg/kg/hr. A 2-ml blood sample was collected through the venous catheter as a control before administration of RMZ. A 2-ml blood sample was collected through the arterial catheter immediately before and after each Pringle maneuver (PM) during surgery. Bilateral red arrows indicated the duration of the PM. Blue arrows indicate the timing of blood sampling. PM, pringle maneuver

The PM technique involved clamping both the hepatic artery and the portal vein for 20 min, followed by unclamping for 5 min, repeated until resection of the tumor was completed. During surgery, a 2-ml blood sample was collected through the arterial catheter immediately before and after each PM (Fig. 1).

### Measurement of RMZ plasma concentrations

Liquid chromatography-tandem mass spectrometry (LC-MS/MS) was performed using QTRAP 4500 LC/MS/MS System (AB Sciex, Framingham, MA, USA) to measure RMZ Cps. Detection was performed by positive electrospray ionization. Plasma samples were obtained by centrifugation of blood at 1,900 × g for 15 min. After adding the internal standard (IS) of filgotinib (25 ng) in methanol (2.5 µL) to a 50-µL sample of patient plasma, 400 µL methanol was added. After vortexing for 30 s and centrifuguring at 16,000 g for 5 min, 2 µL of clear supernatant was injected into the LC-MS/MS. Chromatographic separation was achieved on a reversed-phase Shim-pack Scepter C18-120 (3 μm, 2.1 mm ID × 50 mm, Shimadzu, Kyoto, Japan). The column was eluted with a combination of water (A) and methanol (B) containing 0.1% formic acid at a flow rate of 0.2 mL/min, at an elution gradient of 0–3 min, linear from 60% to 80% B; 3–4 min, 80% B; and 4–5 min, 60% B. RMZ and IS were detected according to the following mass transitions: 439.0 > 362.0 (RMZ) and 426.3 > 291.3 (IS). The primary outcome of this study was to compare RMZ Cps before and after the repeated PMs. In addition, we compared the BIS values before and after the repeated PMs.

### Statistical analysis

To assess the normality of the distribution of the RMZ Cps and BIS values, the Shapiro-Wilk test was used. The test results indicated that the RMZ Cp and BIS values were not normally distributed (*p* < 0.05), justifying the use of nonparametric statistical tests for analysis. To compare RMZ Cps and BIS values, the Wilcoxon signed-rank test and Bonferroni correction were used. P-values less than 0.05 were considered statistically significant. Data are presented as median (IQR, 25–75% interquartile range) and mean ± standard deviation. All statistical analyses were performed by JMP Pro software (ver. 16, SAS Institute, Japan Co., Ltd, Minato-ku, Tokyo.).

## Results

### Successful measurement of RMZ Cp

**Fig. 2:**
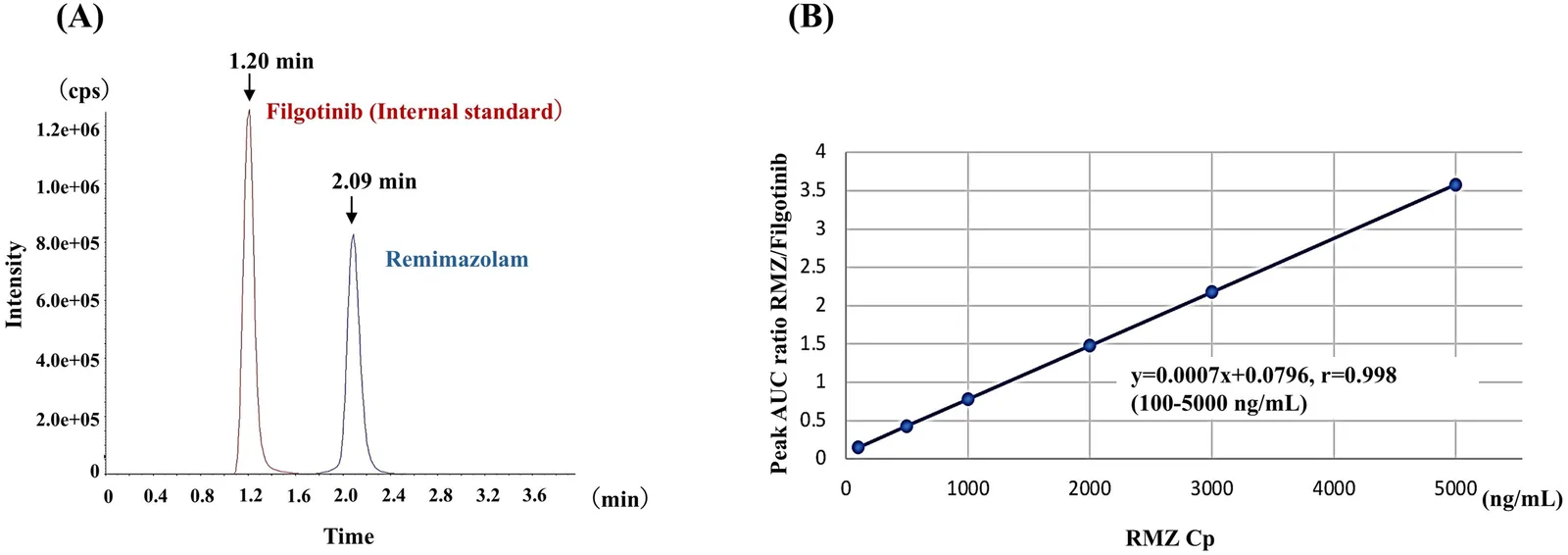
Measurement of plasma concentrations of remimazolam. **A** demonstrates a representative chromatogram of Remimazolam (RMZ) measured from a patient’s sample. Retention times of filgotinib and RMZ were 1.20 and 2.09 min, respectively. **B** illustrates a calibration curve for RMZ in the plasma concentration (Cp) range of 100-5000 ng/mL, generated by the peak area under the curve (AUC) ratio of filgotinib (internal standard) and RMZ. The detection was linear in plasma at RMZ concentrations ranging from 100 to 5,000 ng/mL for RMZ (y = 0.0007 x + 0.0796; r = 0.998). RMZ, remimazolam; AUC, area under the curve; Cp, plasma concentration

Retention times of IS and RMZ were 1.20 and 2.09 min, respectively (Fig. 2A). The detection of RMZ in plasma was linear at RMZ concentrations ranging from 100 to 5,000 ng/mL (y = 0.0007 x + 0.0796; *r* = 0.998, *p* < 0.001) (Fig. 2B).

### Changes in RMZ Cp associated with the PM

**Fig. 3:**
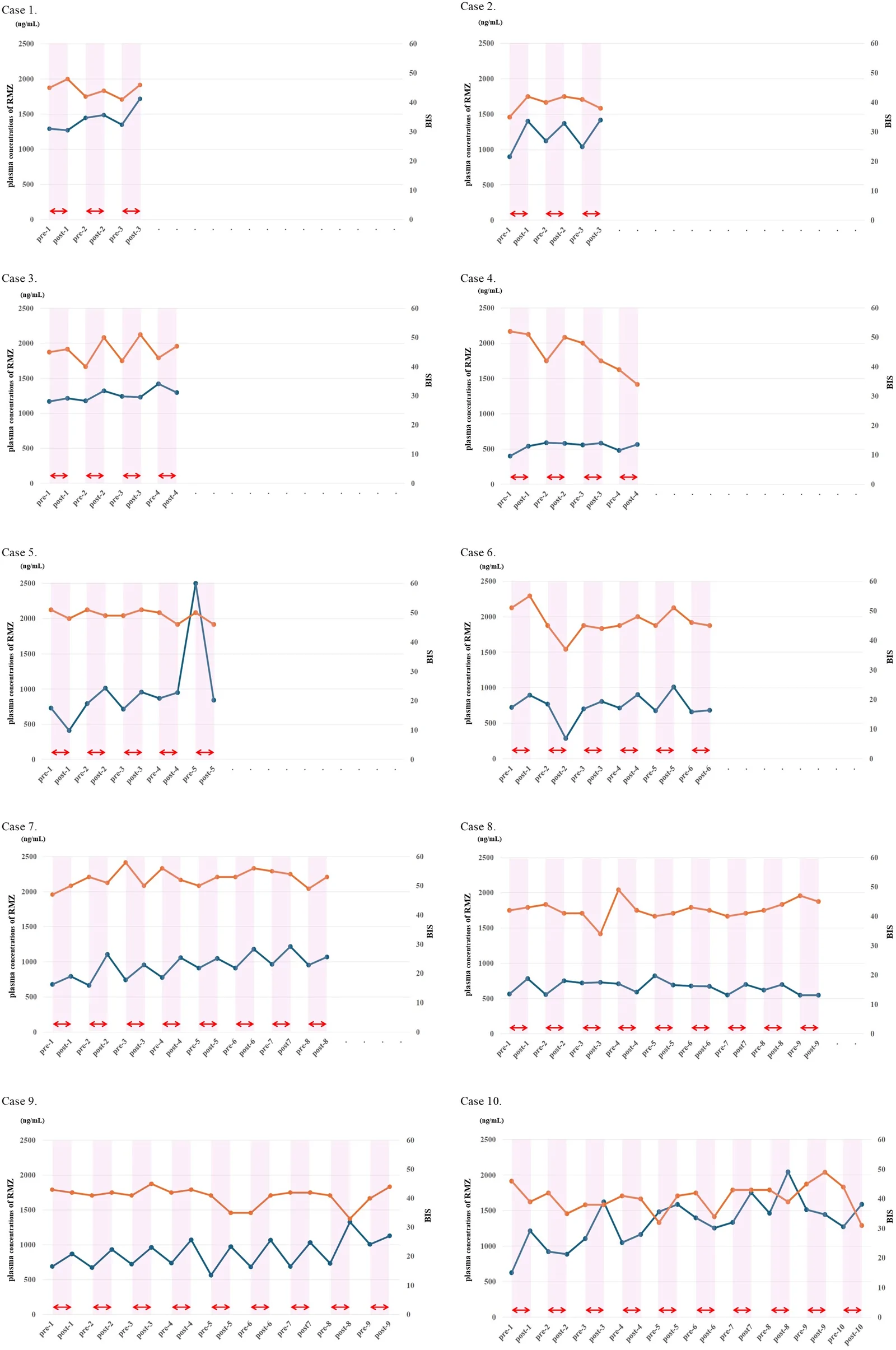
Changes in plasma concentrations of remimazolam and bispectral index during the Pringle maneuvers in each case. The orange lines and blue lines indicate bispectral index values and plasma concentrations of remimazolam, respectively. The pink-colored area and bilateral red arrows indicate the time frame during which the Pringle maneuvers (PM) were conducted

**Fig. 4:**
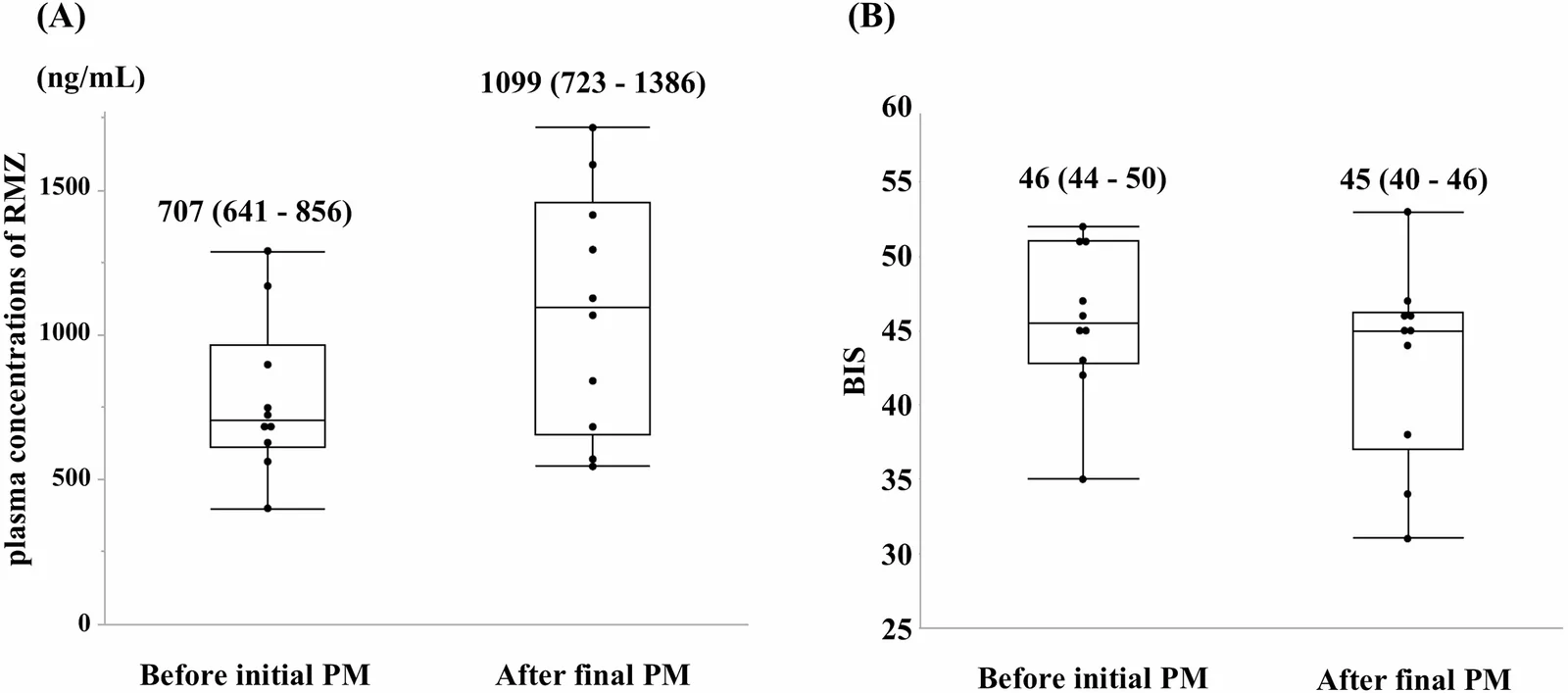
Comparison of plasma concentrations of remimazolam and bispectral index before and after repeated Pringle maneuvers. **A** and **B** depict changes before and after the repeated Pringle maneuvers (PM) in plasma concentrations of remimazolam (RMZ) and bispectral index (BIS), respectively. Neither plasma concentrations of RMZ nor BIS were significantly different before and after the repeated PM. PM, pringle maneuver; BIS, bispectral index

Patient characteristics are summarized in Table 1. All patients had an ASA-PS2 and Child-Pugh classification A. The number of PMs varied due to differences in hepatectomy duration, as demonstrated in Table 1. No cases required additional administration of RMZ, as no prolonged arousal episodes with BIS values exceeding 70 were observed. Figure 3 illustrates the time course of RMZ Cp changes associated with every PM in each case. The changes in RMZ Cp before and after each PM varied depending on the case. RMZ Cp immediately after the PM tended to be higher than those before the PM in six cases (2, 5, 6, 7, 9, and 10), but this was not observed in other cases. Figure 4 demonstrates the changes of RMZ Cps (A) and BIS (B) before and after the repeated PM. There was no statistically significant difference between the median RMZ Cp after the final PM (1099 [723–1386] ng/mL) and prior to initiation of PM (707 [641–856] ng/mL) (*p* = 0.13). Additionally, the median BIS value after the final PM (45 [40–46]) was also comparable to the BIS prior to the initiation of PM (46 [44–50]) (*p* = 0.45). At the end of the surgery, all patients were uneventfully extubated and returned to the general ward without any major postoperative complications.

**Table 1:** Characteristics of patients undergoing hepatectomy

Perioperative patient characteristics	All patients (*n* = 10)
Age, years	66 ± 13 (38–81)
Male	7
Height, cm	161 ± 5 (149–168)
Weight, kg	67 ± 13 (41–83)
BMI, kg/m2	26.0 ± 5.1 (15.6–31.3)
eGFR, ml/min/1.73m2	68.4 ± 14.7 (54.7-107.6)
Child-Pugh classification A	10
ASA-PS 2	10
Type of cancer	
Metastasis	6
Hepatic cancer	3
Intrahepatic bile duct cancer	1
Type of surgery	
Robot assisted surgery	1
Laparoscopic surgery	7
Open	2
Numbers of trials of PM 3/4/5/6/7/8/9/10	2/2/1/1/0/1/2/1
Surgical time, minutes	349.3 ± 109.8
Anesthesia time, minutes	450.7 ± 112.6
Intraoperative fluid volume, ml	2356.5 ± 709.0
Estimated blood loss, ml	421.5 ± 405.5
Urine output, ml	260.0 ± 169.6

Data are expressed as mean ± standard deviation (range), or count. BMI, body mass index; ASA-PS, American Society of Anesthesiologists Physical Status; eGFR, estimated glomerular filtration rate; PM, pringle maneuver

## Discussion

To the best of our knowledge, this is the first study to examine the changes in RMZ Cp associated with the PM used in hepatectomies. Our study showed that overall, RMZ Cp was not significantly affected by repeated PMs. Although RMZ Cp immediately after the PM tended to be higher than those before the PM in six out of ten cases, suggesting that the RMZ Cp increases when the major vessels are clamped during PM, the concern of potential reduction in overall RMZ metabolism is less likely as there was no statistically significant difference between the median RMZ Cp after the final PM and prior to initiation of PM despite the repeated disturbances of blood flow into the liver and reduction in liver volume by surgery. The changes in RMZ Cp immediately following each PM may be explained by the alteration in circulating volume caused by PM secondary to decreased central venous pressure and cardiac output [5–7], possibly related to transient hemodynamic changes during PM. Further, the variation in RMZ Cp changes associated with each PM in different cases may be attributed to the degree of shunt blood flow or the extent of hepatic blood flow interruption caused by the PM. Furthermore, BIS values did not show significant changes during the PMs, indicating that potential alterations of sedative effects are unlikely to be clinically significant. Moreover, we speculate that the lack of significant changes in BIS values may be due to the fact that an adequate depth of anesthesia had already been achieved; therefore, the slight increase in RMZ Cp did not affect the BIS values.

In regards to the severity of hepatic impairment and RMZ Cp, all patients in our study were classified as Child-Pugh A, in which the RMZ Cp was not significantly altered overall following repeated PMs. However, a clinical trial reported that RMZ clearance in subjects with severe hepatic impairment (Child-Pugh classification C) was 38.1% lower compared with healthy volunteers after a single dose of 0.1 mg/kg of RMZ, leading to prolonged recovery to full consciousness (8.0 min in healthy volunteers, 12.1 min with moderate hepatic impairment, and 16.7 min with severe hepatic impairment) [3]. Therefore, investigation of changes in RMZ Cp during the PM in patients with greater hepatic impairment is warranted.

## Conclusions

In conclusion, our study revealed that RMZ Cp and BIS values did not significantly change with repeated PMs during hepatectomy, suggesting that RMZ may be safely administered in patients with Child–Pugh classification A. However, given the limited sample size of this pilot study, further large-scale studies are warranted to confirm these findings, to elucidate the potential impact of PM-related changes in RMZ Cp on BIS values, and to determine the optimal dosing of RMZ during PM, particularly in patients with more severe hepatic impairment.

## Data Availability

The datasets used and analyzed during the current study are available from the corresponding author on request.

## Abbreviations

ASA-PS: American Society of Anesthesiologists Physical Status
AUC: Area under the curve
BIS: Bispectral index
CES1: Carboxylesterase 1
Cp: Plasma concentration
eGFR: Estimated glomerular filtration rate
IS: Internal standard
IQR: Interquartile range
LC-MS/MS: Liquid chromatography–tandem mass spectrometry
PM: Pringle maneuver
RMZ: Remimazolam
SD: Standard deviation
